# Bone fragility in sarcoidosis and relationships with calcium metabolism disorders: a cross sectional study on 142 patients

**DOI:** 10.1186/ar4519

**Published:** 2014-03-24

**Authors:** Nathalie Saidenberg-Kermanac’h, Luca Semerano, Hilario Nunes, Danielle Sadoun, Xavier Guillot, Marouane Boubaya, Nicolas Naggara, Dominique Valeyre, Marie-Christophe Boissier

**Affiliations:** 1 INSERM UMR1125, Bobigny, France; 2 Sorbonne Paris Cité-Université Paris 13, Bobigny, France; 3 Assistance Publique-Hôpitaux de Paris, Groupe Hospitalier Avicenne-Jean Verdier-René Muret, Department of Rheumatology, Bobigny, France; 4 Assistance Publique-Hôpitaux de Paris, Groupe Hospitalier Avicenne-Jean Verdier-René Muret, Department of Respiratory Diseases, Bobigny, France; 5 Assistance Publique-Hôpitaux de Paris, Groupe Hospitalier Avicenne-Jean Verdier-René Muret, Clinical Research Unit, Bobigny, France; 6 Assistance Publique-Hôpitaux de Paris, Groupe Hospitalier Avicenne-Jean Verdier-René Muret, Department of Radiology, Bobigny, France

## Abstract

**Introduction:**

The prevention of fragility fractures in patients with sarcoidosis is a serious concern and the potential risk of hypercalcemia limits vitamin D and calcium supplementation. The objective of this study was to evaluate the risk factors for low bone mineral density (BMD) and fractures in sarcoidosis. In particular, we aimed to determine the link among bone fragility and calcium and vitamin D metabolism in this population.

**Methods:**

We performed a cross-sectional analysis on 142 consecutive patients with histologically proven sarcoidosis. BMD and prevalence of vertebral fractures on X-rays were assessed and the association with potential risk factors was studied by regression analysis.

**Results:**

Fragility fractures occurred in 23.5% of patients, despite a normal mean BMD in the study population. In a multivariate analysis, low dietary calcium, fracture, age, gender and menopause were associated with increased risk of low BMD. Low dietary calcium, high current corticosteroid dose and low creatinine clearance were associated with increased risk of fracture. Serum 25(OH)D between 10 and 20 ng/ml was significantly associated with higher BMD. Conversely, values greater than 20 ng/ml were associated with increased risk of fracture. Serum 25(OH)D level was inversely correlated with disease activity. Of note, vitamin D supplements increased serum 25(OH)D in a dose-dependent manner but had no effect on serum calcium level.

**Conclusions:**

Sarcoidosis patients have a high risk of fracture despite not having a lowered BMD suggesting that other independent factors are involved. Current corticosteroid dose, low dietary calcium and serum 25(OH)D levels are associated with bone fragility. In sarcoidosis, calcium and vitamin D supplementation might be warranted, but desirable 25(OH)D serum levels might be lower than those advised for the general population.

## Introduction

Sarcoidosis is a multifaceted granulomatous disease ranging from regressive localized forms to chronic systemic involvement. The risk of fracture in sarcoidosis has not been clearly evaluated although most patients may present risk factors of osteoporosis. Like in other chronic diseases, as rheumatoid arthritis (RA) [1] or spondyloarthritis [2], both corticosteroid (CS) use and systemic inflammation could promote bone loss in sarcoidosis.

Disorders of calcium and vitamin D metabolism could also interfere with bone mineral density in sarcoidosis. Extra renal synthesis of the active form of vitamin D (1,25(OH)2D) takes place inside the granulomas under the influence of 1alpha-hydroxylase. In contrast to the renal enzyme, the 1-alpha-hydroxylase expressed by macrophages is not inhibited by serum 1,25(OH)2D levels; moreover, the stimulation of the 25(OH)D-24-hydroxylase transforming the 1,25(OH)2D into inactive 24,25(OH)2D is stimulated only at very high levels of 1,25(OH)2D [3,4]. The resulting high levels of calcitriol could contribute to increased intestinal absorption of calcium, which might partly explain the hypercalcemia sometimes observed in sarcoidosis. Moreover, sarcoidosis patients are more sensitive than healthy subjects to vitamin D supplements with a higher increase in serum calcium after intake [5]. For these reasons, many experts advise patients to avoid sun exposure and vitamin D and calcium supplements, with the potential risk of chronic vitamin D deficiency.

These risks factors are nevertheless balanced by the fact that the disease evolves generally in young adults, at lower risk of fracture. Moreover, CS-free remission periods can be very long, and it was shown that in sarcoidosis the effect of CS on bone might be, at least partially, reversible [6,7]. Few studies with contradictory results are available in the literature, probably due to limited sample size and heterogeneous clinical presentation of included patients. Previous studies using quantitative computed tomography (QCT) showed a reduction of bone mineral content (BMC) even in patients not treated with CS [7,8]. Thereafter, similar results were found only in lumbar spine BMD of post-menopausal women [9] or in CS-treated subjects [10-12]. In contrast, in a four-year longitudinal study, no bone loss was observed even in CS-treated patients despite a high rate of fracture (38%) observed by vertebral fracture assessment (VFA) [13]. This suggests that other determinants apart from BMD are probably involved in fracture risk. So far, the link between serum 25(OH)D level and BMD has not been studied in sarcoidosis.

Our objective was to determine the risk factors for bone fragility evaluated by BMD and fracture prevalence in sarcoidosis patients and in particular to evaluate the relationship with vitamin D and calcium metabolism in a pilot cross-sectional study.

## Methods

### Study design

We included 142 consecutive patients with sarcoidosis according to the criteria retained in the Consensus Conference ATS/ERS [14], that is, combining clinical, biological and radiological presentation compatible with diagnosis and excluding other granulomatous diseases. Among 222 patients attending pneumonology consultation or day-hospital during the inclusion period, 74 were excluded because they did not meet inclusion criteria and 6 refused to participate in the study. All 142 remaining included patients presented documented histological lesions of granuloma without caseous necrosis, in at least one site of biopsy. Patients with other chronic progressive diseases, chronic respiratory or renal insufficiency stage IV and V whose origin was not related to sarcoidosis and those on diuretics able to interfere with calcium metabolism were excluded from the study. At inclusion, all patients underwent clinical examination evaluating the risk factors for osteoporosis and calcium intake. All patients had biochemical, radiological and BMD assessment.

This study complies with the Declaration of Helsinki and was approved by the Ethical Committee of France 10 (N° ID RCB 2011-A00202-39). All patients gave their informed consent prior to their inclusion in the study.

### Clinical parameters

For each patient, a questionnaire assessed the following risk factors for osteoporosis: age, sex, menopausal status, tobacco and alcohol consumption, body mass index (BMI), personal or family history of fracture of low energy (defined as resulting from a fall from standing height or lower; skull, metacarpal and metatarsal fractures were excluded).

Anti-osteoporotic treatments, current and cumulated CS dose and vitamin D supplements in the six months preceding the study were recorded. Dietary calcium intake was evaluated by the Fardellone auto-questionnaire [15].

The data related to sarcoidosis were also collected: disease duration, localization (graded from 0 to 6 according to the degree of severity of each organ involvement), current flare (at least one active localization or new localization in the three months preceding the study), number of relapses, stage of pulmonary involvement (stage 0: normal chest radiography; stage I: bilateral hilar lymphadenopathy without pulmonary infiltrates; stage II: bilateral hilar lymphadenopathy with pulmonary infiltrates; stage III: pulmonary infiltrates without bilateral hilar lymphadenopathy; stage IV: end-stage fibrosis, bullae, honeycombing and cavity), stage of dyspnoea from I to IV according to New York Heart Association (NYHA) classification.

### Biochemical parameters

The serum levels of calcium (corrected with albumin), phosphate, 25(OH)D (Diasorin radioimmunoassay, Stillwater, US), 1,25(OH)2D (IDS radioimmunoassay, Frankfurt, Germany), creatinine, parathyroid hormone (PTH) (immunometry LIA, Immulite 2000, Puteau, France), thyroid stimulating hormone (TSH), bone markers: bone alkaline phosphatases (BALP) (ELISA, reactif microrevue), C-terminal telopeptide of type I collagen (CTX) (ECLIA/Cobas-Roche, Switzerland), osteocalcin (immunometry TRACES, Kryptor), 24-hour urinary calcium, phosphate and creatinine, were gathered. Other parameters: erythrocyte sedimentation rate (ESR), C-reactive protein (CRP), serum angiotensin-converting enzyme (ACE) level, blood count and serum albumin level were also gathered.

### Imaging parameters

Radiographs of thoracic and lumbar spine (face and profile) were carried out. Assessment of vertebral fractures was independently performed by both a radiologist (NN) and a rheumatologist (NSK) according to the semi-quantitative method of Genant [16] and their respective results were blinded during assessment. Inter-rater reliability was good K = 0.78, 95% CI (0.63 to 0.93). Vertebral fracture was defined by reduced height loss above 20% of the mean, posterior or anterior wall.

BMD was measured by dual X-ray absorptiometry (DXA Lunar Prodigy, GE Healthcare) at the lumbar spine and the total hip. Osteopenia and osteoporosis were defined by a-1SD < T-score < -2.5 SD and T-score ≤ -2.5 SD, respectively. Low BMD was defined by a T-score < -1 SD (osteopenia or osteoporosis).

### Statistical analysis

Data are summarized as the mean and standard deviation for continuous data and frequency for categorical data. A logistic regression model was used to identify factors associated with low BMD (defined as a T-Score lower than -1 SD) and risk of fracture. 25(OH)D was grouped in three categories as retained by the Institute of Medecine [17]: ≤10 ng/ml (deficiency), 10 to 20 ng/ml (insufficiency) and ≥20 ng/ml (desirable). All factors with *P* <0.20 at univariate analysis were included in a multiple logistic regression model with backward selection. Age, gender and menopause were grouped together to avoid a colinearity problem in multivariate analysis. Serum 25(OH)D and calcium levels according to different criteria were compared with the Mann-Whitney U-test, with a Bonferroni correction for multiple tests. The associations between the continuous factors were determined with Spearman’s correlation coefficients. All tests were two-sided at a 0.05 significance level. Analyses were carried out using R statistical software version 2.14.1.

## Results

### Clinical characteristics of the patients

One hundred forty two patients, 80 women (51 menopaused) and 62 men, were included. Eighty-five patients were Caucasians, 54 Caribbeans and 3 Indians. Mean age was 51.6 ± 11.6 years and BMI 27.5 ± 5.4. Mean disease duration was 9.5 ± 7.1 years and 104 patients had presented more than one relapse; 21/137 patients experienced a low energy fracture before inclusion. Eighty-eight patients were receiving CS treatment at the time of the study (mean dose 12.4 ± 11.8 mg/d of prednisone equivalent), 28 patients had never received CS, 45 interrupted CS treatment for at least six months. The mean cumulated CS dose was 27.6 ± 19.9 g of prednisone equivalent. Thirty-one patients had received vitamin D supplements in the six months preceding the study (mean dose 181,161 ± 137,430 U) and 15 were on supplementation at inclusion. Forty-six patients had received a specific bone treatment before the study (bisphosphonates in the majority) on average for 25.3 ± 26.7 months; 24 were still treated during the study. The mean daily dietary calcium was 717.2 ± 360 mg (Table 1).

**Table 1:** Patients characteristics and biochemical parameters

Women/men (number of patients)	-80/62
Menopausal women (number of patients)	51/80
Age (years)	51.6 ± 11.6
Mean disease duration (years)	9.5 ± 7.1
BMI (mean ± SD)	27.5 ± 5.4
Radiological Stage I/ II/ III/IV/ (number of patients)	15/69/14/41
Dyspnoea Stage NYHA I and II/ NYHA III and IV (number of patients)	118/45
History of low energy fracture (number of patients)	21
Current CS intake (mean dose): 88 patients	12.4 ± 11.8 mg
Mean cumulative CS dose per patient	27.6 ± 19.9 g
Vitamin D supplements (mean dose) in the six months before the study: 31 patients	181,161 ± 137,430 U
BP treatment (mean treatment duration) before study: 46 patients	25.3 ± 26.7 months
Mean serum 25(OH)D level (N >30 ng/ml)	14.5 ± 7.61
Mean serum 1,25(OH)2D level (66 < N <167 pmol/l)	137.4 ± 50.3
Mean serum PTH level (10 < N <70 pmol/l)	45.36 ± 31.4
Mean creatinine clearance (ml/min)	110.7 ± 35.9
Mean serum calcium level (2.2 < N <2.6 mmol/l)	2.35 ± 0.9
Mean ESR (mm/h)	17.6 ± 14.5
Mean CRP (mg/L)	7.0 ± 9.8
Mean ACE (19 < N <41)	51.7

ACE, angiotensin-converting enzyme; BMI, Body mass index; BP, bisphosphonates; CRP, C-reactive protein; CS, Corticosteroids; ESR, erythrocyte sedimentation rate; NYHA, New York Heart Association; PTH, parathyroid hormone.

### Biochemical parameters of bone metabolism

Biochemical parameters are shown in Table 1. Serum calcium was within the normal range with no significant difference between summer (June to August) and winter (December to February), respectively, with a mean level of 2.36 ± 0.06 mmol/l vs 2.32 ± 0.1 mmol/l ( *P* = 0.16). Only one patient had high serum calcium (>2.6 mmol/L), (without hypercalciuria) related to a systemic form with documented bone sarcoidosis lesions. Nine out of 94 patients presented hypercalciuria (24-hour urinary calcium >0.1 mmol/kg).

**Figure 1:**
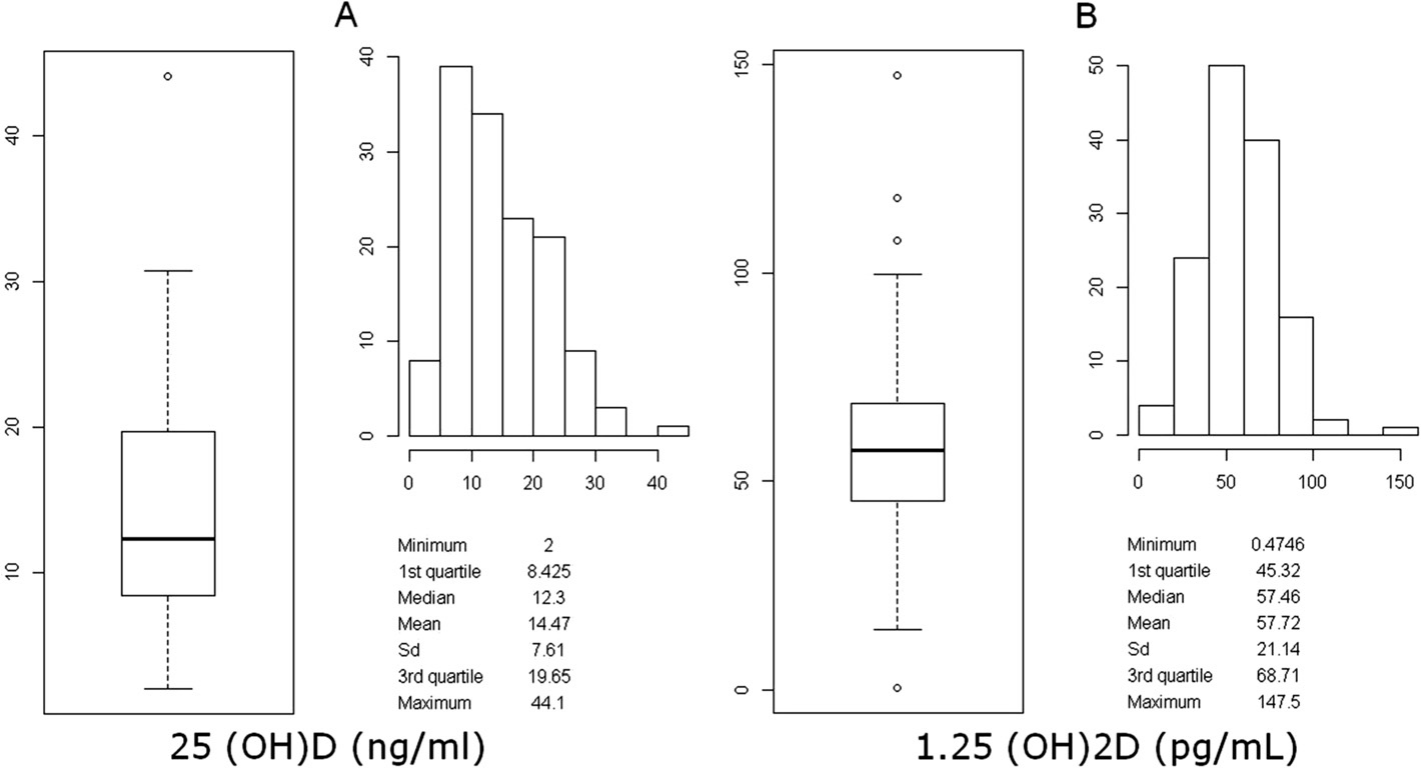
Distribution of 25(OH)D (A) and 1,25(OH)2D (B) serum level in the study population.

**Figure 2:**
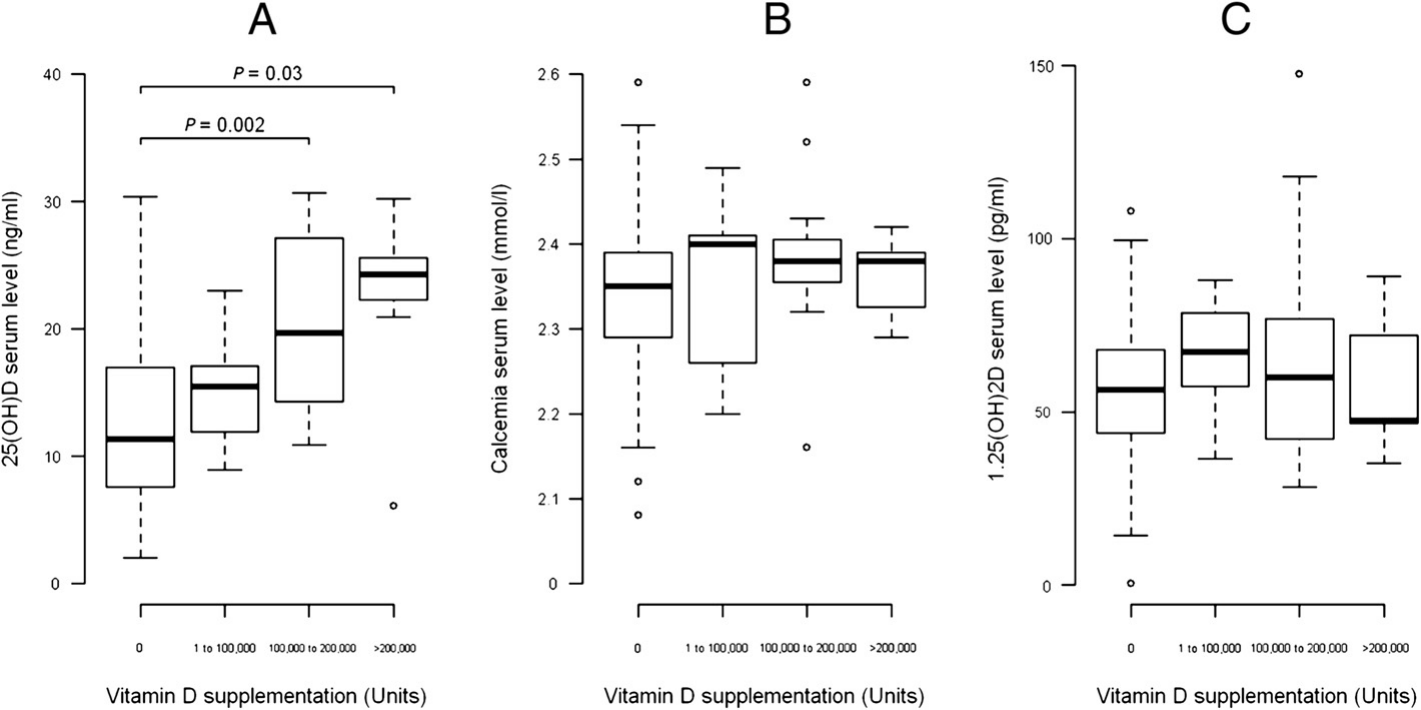
**Changes in serum 25(OH)D, calcium and 1,25 (OH) 2D level after vitamin D supplementation.** Changes in serum 25(OH)D **(A)** calcium **(B)** and 1,25 (OH) 2D **(C)** level according to the dose of vitamin D supplementation in the six months that preceded the study. There is no significant change in calcium and 1,25 (OH) 2D serum level depending on the total amount of vitamin D supplementation.

Serum 25(OH)D levels were low (Figure 1) with a mean of 14.5 ± 7.61 ng/ml but with normal mean PTH serum level. The 31 patients who had received vitamin D supplements had significantly higher 25(OH)D but not higher serum calcium levels vs. those not supplemented and no significant change in 1,25(OH)2D serum level (Figure 2).

CS-treated or untreated patients did not differ in 25(OH)D and 1,25(OH)2D serum levels nor in CRP or ESR (trend toward a lower ESR on CS).

There was no association between bone markers and BMD value or fracture. However, as expected, CS-treated patients had significantly lower bone marker serum levels: (CTX : 372.1 pg/ml with CS vs. 480.6 pg/ml without CS, *P* = 0.01; BALP : 30.0 ± 13.5 UI/L vs 33.5 ± 10.8 UI/L, *P* <0.05; osteocalcin: 15.5 ± 10.1 ng/ml vs 25.2 ± 13.2 ng/ml, *P* <0.001).

Vitamin D supplementation was positively correlated with serum 25(OH)D (r = 0.38, *P* <0.001) but not with serum calcium level.

Serum levels of 25(OH)D show significant but weak inverse correlation with disease flares (r = -0.18, *P* <0.05), the severity of the pulmonary involvement (r = -0.18, *P* <0.05), ACE (r = -0.19, *P* <0.05) and ESR (r = -0.19, *P* <0.05). 1,25(OH)2D serum levels are positively correlated with serum 25(OH)D (r = 0.35, *P* <0.001) and with no other parameter. In particular, we did not find correlation between 1,25(OH)2D serum level and chronicity of the disease (r = -0.08, *P* = 0.37) that was found by D Kavathia *et al*. [18] or the number of relapses.

### Osteoporosis prevalence in sarcoidosis

The mean BMD was normal: Tscore: -0.5 SD at the lumbar spine and -0.09 SD at the total hip. However, 34.8% presented low BMD at the lumbar spine (37 patients had osteopenia and 11 patients had osteoporosis, respectively) and 24% had osteopenia or osteoporosis at the hip (24 patients and 9 patients, respectively). On the whole, 40.15% (55/137) were osteopenic at one skeletal site at least, and 14.6% (20/137) were osteoporotic.

A total of 13.6% of the patients (19/139) had at least one vertebral fracture and 10 patients presented with two or more vertebral fractures. Overall, 23.5% of patients (32/136) had at least one vertebral or peripheral fracture.

### Risk factors for bone fragility in sarcoidosis

Table 2 shows the odds ratio of low BMD and fractures at univariate analysis. Low BMD (T-score < -1 SD) is associated with age, menopause, prevalent fracture, low dietary calcium intake, cumulative CS dose, long disease duration, advanced-stage dyspnoea (III or IV), lymphopenia, high ESR and low creatinine clearance. The patients with serum levels of 25(OH)D between 10 and 20 ng/ml have the lowest odds of low BMD, whereas the odds increase when this threshold is exceeded. Fractures were significantly associated with age, low dietary calcium intake, cumulative and current CS dose, advanced-stage dyspnoea (III or IV), low creatinine clearance and low BMD. Of note, 25(OH)D levels exceeding 20 ng/ml are associated with significantly higher odds of fracture (Table 2). BMI, ethnicity, type of sarcoidosis involvement, CS-free period duration, smoking, 1,25(OH)2D serum level, CRP, bone remodelling markers showed no association with BMD or fracture.

**Table 2:** Factors associated with low BMD and fracture at univariate analysis

	**BMD < −1SD**		**Fracture**	
	**OR [95% CI]**	***P***	**OR [95% CI]**	***P***
Menopause	5.29 [1.83; 15.27]	0.002	2.1 [0.68; 6.53]	0.2
Age, years	1.06 [1.03; 1.1]	<0.001	1.09 [1.05; 1.14]	<0.001
Calcium intake <500 mg/d	4.28 [1.89; 9.72]	<0.001	2.2 [0.93; 5.18]	0.072
Stage NHYA 3 or 4	3.34 [1.3; 8.54]	0.012	3.18 [1.23; 8.2]	0.017
Disease duration	1.05 [1; 1.1]	0.047	1 [0.95; 1.06]	0.9
Cumulative CS dose	1.15 [0.98; 1.36]	0.088	1.2 [0.99; 1.44]	0.059
Current CS treatment	1.68 [0.83; 3.4]	0.15	2.63 [1.08; 6.37]	0.033
Vitamin D supplements	3.82 [1.66; 8.8]	0.002	2.07 [0.87; 4.95]	0.1
BP treatment	3.03 [1.44; 6.36]	0.003	1.83 [0.8; 4.15]	0.15
ESR >10 mm/h	2.49 [1.1; 5.62]	0.028	1.38 [0.55; 3.46	0.5
Lymphocytes >1,000/mm^3^	0.45 [0.21; 0.94]	0.033	1.19 [0.5; 2.85]	0.7
Creatinine clearance, ml/mn	0.99 [0.98; 1]	0.034	0.97 [0.95; 0.99]	<0.001
25(OH)D serum level				
25(OH)D ≤10 ng/ml	1	-	1	-
10 < 25(OH)D <20 ng/ml	0.48 [0.21; 1.09]	0.079	1.24 [0.45; 8.23]	0.67
25(OH)D >20 ng/ml	2.00 [0.8; 4.97]	0.13	2.94 [1.05; 8.23]	0.04
Serum PTH level, pmol/l				
Fracture/low BMD	4.39 [1.86; 10.37]	<0.001	4.39 [1.86; 10.37]	<0.001

BMD, bone mineral density; BP, bisphosphonates; CS, Corticosteroids; ESR, erythrocyte sedimentation rate; PTH, parathyroid hormone; NYHA, New York Heart Association.

We then assessed factors associated with low BMD using multivariate analysis (Table 3). Age, prevalent fracture, female gender, menopause, low dietary calcium, lymphopenia and vitamin D supplementation are associated with higher odds of low BMD. Again, the patients with 25(OH)D serum levels between 10 and 20 ng/ml are at lower risk of low BMD vs. those with levels <10 ng/ml (reference class), while levels exceeding 20 ng/ml are associated with a higher risk of fracture. Low dietary calcium and high CS doses are also associated with a higher risk of fracture.

**Table 3:** Factors associated with low BMD and fracture at multivariate analysis

	**BMD < −-SD**		**Fracture**	
	**OR [95% CI]**	***P***	**OR [95% CI]**	***P***
Menopausal female	13.84 [2.28; 84.11]	0.004		
Male ≥50 years	12.2 [1.42; 104.71]	0.023		
Calcium intake <500 mg/d	3.98 [1.19; 13.25]	0.025	3.5 [1.09; 11.27]	0.036
Vitamin D supplements	12.86 [2.98; 55.53]	0.001		
BP treatment	4.6 [1.47; 14.3 ]	0.009		
Fracture	3.88 [0.99 ;15.23]	0.052		
25(OH)D ≤10 ng/ml	1	-	1	-
10 < 25(OH)D ≤20 ng/ml	0.29 [0.08; 1.06]	0.062	2.05 [0.57; 7.45]	0.274
25(OH)D >20 ng/ml	0.96 [0.25; 3.67]	0.95	3.93 [1.02; 15.17]	0.047
Current CS treatment			3.73 [1.06; 13.16]	0.04
Creatinine clearance			0,97 [0.94; 0.99]	0.002

BMD, Bone mineral density; BP, Bisphosphonate; CS, Corticosteroids. Empty cells correspond to variables not included in the multivariate analysis because they were not significant at univariate analysis or to variables included in the multivariate analysis but not retained in the final model because they were no longer significant (for example, age).

**Figure 3:**
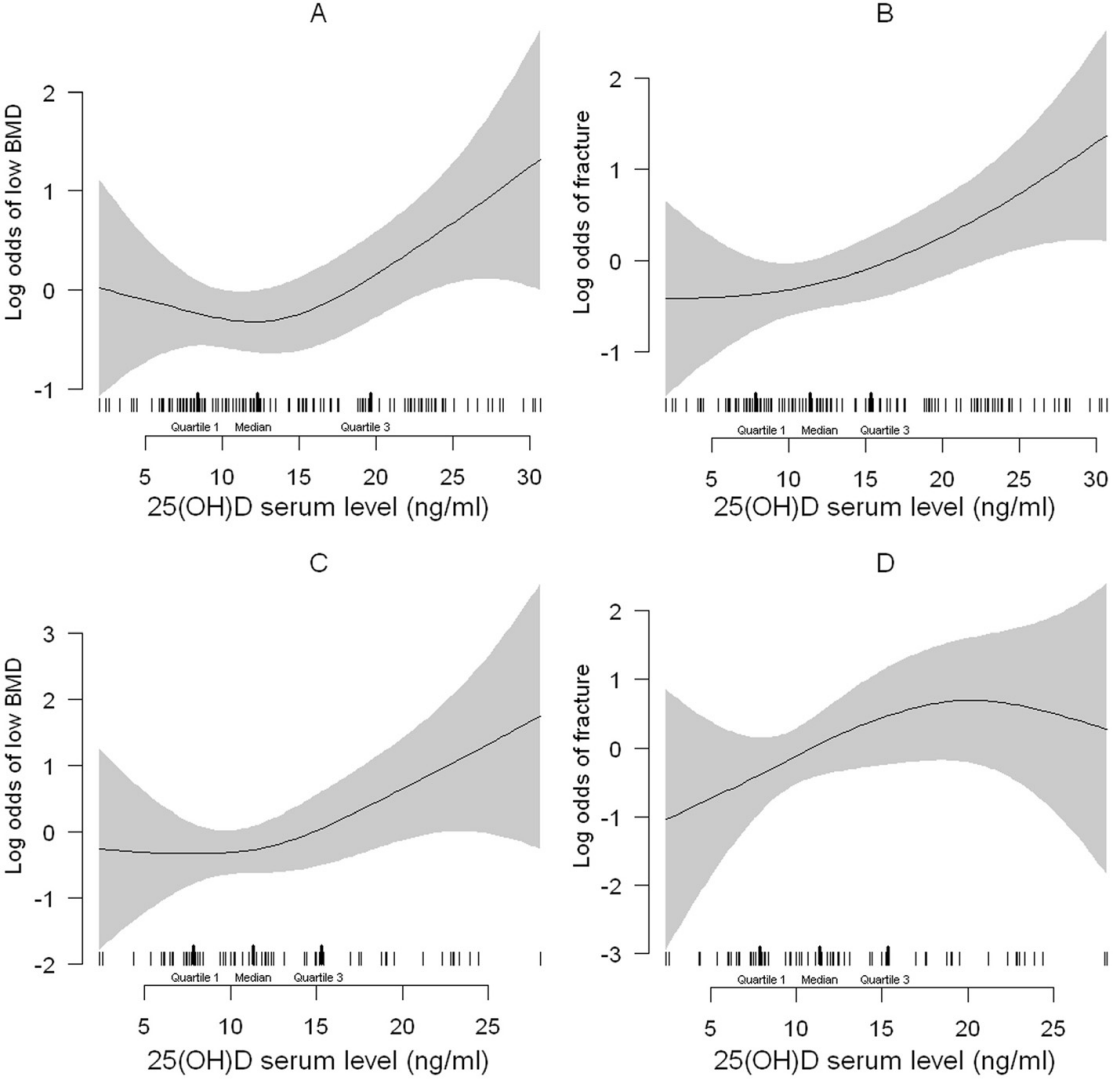
**Relation between 25(OH)D and low BMD and fracture.** Relation between 25(OH)D (ng/ml) and the odds of low BMD (**A**, **C**) and fracture (**B**, **D**) with the use of a generalized additive model (GAM) for the totality of patients (**A**, **B**) and after exclusion of Vitamin D or BP-treated patients (**C**, **D**) respectively. The relationship between vitamin D levels and the odds of low BMD follows a U-shaped curve (**A**, **C**) while the relationship with fracture risk follows a linear relationship. Shaded areas represent the 95% confidence intervals and the tick marks show the distribution (with median and interquartile range) of 25(OH)D values. BMD, bone mineral density; BP, bisphosphonates.

The relationship between 25(OH)D serum level and risk of low BMD in our population, using a generalized additive model followed a U-shaped curve: patients with serum 25(OH)D levels between 10 and 20 ng/ml are at lower risk of low BMD. The relationship between 25(OH)D and fracture is more linear, but with a steeper slope for 25(OH)D values above 20 ng/ml (Figure 3A, B).

We then evaluated whether history of vitamin D supplementation or BP treatment modified the relationship between 25(OH)D serum level, low BMD and fracture. In our sample, vitamin D-supplemented or BP-treated patients had higher prevalence of bone fragility fracture (*P* <0.01) and lower BMD at both the lumbar spine (*P* <0.01) and hip (*P* <0.05) (Table 4).

**Table 4:** Prevalence of patients having low BMD (≤1 SD) or at least one prevalent fracture according to 25(OH)D serum levels

	**BMD**		**Fracture**	
	**All patients**	**No BP or Vitamin D**	**All patients**	**No BP or Vitamin D**
25OHD <10	20/46 (43%)	8/29 (27%)	8/46 (17%)	2/27 (7%)
10 < 25OHD <20	15/56 (27%)	5/31 (16%)	11/53 (21%)	8/28 (28%)
25OHD >20	20/33 (61%)	4/7 (57%)	13/34 (38%)	2/7 (28%)

BMD, bone mineral density; BP, bisphosphonates. The results are reported for the whole sample and for those patients that had not received BP treatment or vitamin D supplementation.

The relationship between 25(OH)D serum level and BMD is unmodified if those patients are excluded from the analysis (interaction between 25(OH)D serum level and vitamin D supplements or BP treatment, *P* = 0.71), (Figure 3C). Conversely, as far as fracture is concerned, there is a significant interaction between 25(OH)D and vitamin D and BP treatment (*P* <0.05), explained by the low prevalence of fracture in patients with serum levels of 25(OH)D <10 ng/ml if vitamin D and BP-treated patients are excluded (Table 4). Nevertheless, even after exclusion of these patients, the relationship between 25(OH)D levels and fracture remains linear (Figure 3D).

## Discussion

This study is the first that assesses the link between the metabolism of calcium and vitamin D and the risk of osteoporosis in a population of patients with sarcoidosis. We observe that 25(OH)D levels are associated with low BMD and fracture and might be a risk factor for both. In addition, we found a high prevalence of fracture contrasting with a normal mean BMD in this population. The results of this study allow us to highlight three significant points concerning vitamin D in sarcoidosis.

First, we found a significant association among 25(OH)D serum level, BMD and risk of fracture. Levels ranging between 10 and 20 ng/ml are associated with higher BMD while this association is lost for higher values, which are conversely associated with higher risk of fracture. These associations do not seem to be due to vitamin D supplement in patients with lower BMD or higher risk of fracture. In fact, the associations persisted at multivariable analysis after correction for vitamin D supplementation in the last six months. Moreover, the exclusion of patients having received vitamin D or BP supplements to prevent corticosteroid-induced osteoporosis did not affect the relationship between 25(OH)D and BMD or fracture at generalized additive model analysis. These data suggest that excessive vitamin D supplement could be deleterious in these patients.

This notion might be supported by the results of recent studies on the general population: Vital D Study [19], a double-blind, randomized, controlled trial involving 2,317 community-dwelling women (mean age 70 years) randomly assigned to receive either a single oral dose of cholecalciferol 500,000 IU or placebo yearly for three to five years, found a higher risk of fracture and fall in supplemented women in whom baseline 25(OH)D serum level increased from 19.6 to 48.07 ng/ml. Grimnes *et al*. [20] found that excessive vitamin D supplementation (inducing mean maximum serum level 74 ng/ml) is associated with lower BMD and decrease of bone remodeling in osteopenic post-menopausal women. Finally, Ensrud *et al*. [21] found that association between 25(OH)D and frailty status (a risk factor of fall and fracture) may have a U-shaped pattern with increasing odds of frailty at the lower (<20 ng/ml) and higher (≥30 ng/ml) 25(OH)D levels.

In sarcoidosis patients, known to be more sensitive to vitamin D [5], the optimal range of 25(OH)D levels might be lower than that desirable for the general population as it has been described in idiopathic infantile hypercalcemia. In this rare disease, the presence of *CYP24A1* mutation causes inactivation of 24-hydroxylase and explains the increased sensitivity to vitamin D [22]. In this disease, 25(OH)D serum must be maintained at low levels to avoid hypercalcemia.

Even if the biological mechanism of the toxicity of high vitamin D levels remains speculative [23-25], extrarenal synthesis of 1.25(OH)2D in sarcoid granuloma resulting in excessive 1.25(OH)2D levels, could be involved [3]. In our sample, there was a positive correlation between 25(OH)D and 1,25(OH)2D serum levels. While physiological levels of 1.25(OH)2D inhibit PTH-dependent bone resorption, higher levels are conversely known to induce bone resorption. Hamada *et al*. [26] found that in female (but not male) CS-free sarcoidosis patients, 1.25(OH)2D levels were negatively correlated with lumbar Z-score and serum calcium and positively correlated with osteocalcin. In our patients, whether treated with CS or not, we did not find any correlation between serum 1,25(OH)2D and bone markers apart from a negative correlation with bone alkaline phosphatase. However, dosing of 1.25(OH)2D is subject to fluctuations [27], and our population was not homogeneous: the patients had variable forms of the disease and most of them were on CS. All these factors might have interfered with the results. Nevertheless, consensus exists that the level of 25(OH)D (precursor of 1,25(OH)2D) is more stable and more relevant to evaluate vitamin D status [28]. Hence, 25(OH)D might be better suited to assess the relationship between vitamin D and both osseous and extra-osseous involvement of the disease.

The second point concerning vitamin D is the possible correlation between low 25(OH)D serum level (and not 1.25(OH)2D) and the parameters of disease activity. This was suggested in other inflammatory diseases, such as RA, where low 25(OH)D levels were associated with disease flares [29,30].

The third important point is that patients supplemented with vitamin D have significantly higher serum 25(OH)D but not higher serum calcium vs. non-supplemented patients. Accordingly, Adler *et al*. [10] did not find any impact of calcium and vitamin D supplementation on serum or urinary calcium in sarcoidosis patients.

Overall, our findings suggest that vitamin D supplementation should be considered in sarcoidosis patients but should probably target a threshold that might be lower than that advised for the general population. In addition, low dietary calcium correlated with low BMD and high risk of fracture support the need for adequate calcium intake in these patients.

Nevertheless, the risk of hypercalcemia after vitamin D supplementation reported in other series should lead to caution [31]. Further studies are needed to better identify what patient can be safely supplemented in calcium and Vitamin D and at what dose.

We observed a higher prevalence of fracture compared to epidemiological data on healthy adults of the same age [32]. This prevalence is also close to that found in young, adult CS-treated patients with other diseases [33] and to that found by Heijckmann [34] in a cross-sectional study in sarcoidosis patients. Even if low BMD was correlated with the risk of fracture, mean BMD was normal in our study patients, contrasting with the high prevalence of fracture. This suggests that other factors than BMD are involved, and must be taken into account, in the evaluation of global fracture risk in these patients. Among them, cumulative CS dose, age, respiratory insufficiency and altered renal function were all associated with increased risk of fracture at univariable analysis.

We did not find any correlation between BMD or fractures and parameters of inflammation but in this study the average ESR and CRP were low. However, lymphocyte count was inversely correlated with BMD at both univariable and multivariable analysis.

The main study limitations lie in the cross-sectional and monocentric design, in the lack of a control population and the heterogeneity of the disease profile in the study population. This last point is difficult to prevent to get a sufficient sample size of patients but the multivariate analysis allows us to identify the main risk factors of osteoporosis. The main strength is the large sample size for this disease. These preliminary data need to be confirmed in longitudinal studies in particular to verify the association between the different levels of 25(OH)D and the risk of fracture or low BMD.

## Conclusion

This is the first study that establishes a link between vitamin D levels and bone mineral density in patients with sarcoidosis and suggests an optimal threshold of 25(OH)D in this population. Furthermore, these data suggest that particular risk factors for osteoporosis should be taken into account for sarcoidosis patients, whose fracture risk is high and poorly related to BMD.

## Abbreviations

ACE: Angiotensin-converting enzyme; BALP: Bone alkaline phosphatases; BMD: Bone mineral density; BMI: Body mass index; BP: Bisphosphonate; CRP: C-reactive protein; CS: Corticosteroids; CTX: C-terminal telopeptide of type I collagen; ESR: Erythrocyte sedimentation rate; NYHA: New York Heart Association; PTH: Parathyroid hormone; QCT: Quantitative computed tomography; RA: Rheumatoid arthritis; TSH: Thyroid stimulating hormone; VFA: Vertebral fracture assessment.

## Competing interest

All authors state that they have no conflicts of interest.

## Authors’ contribution

NSK worked on the study conception and design, study conduct, data collection and analysis, data interpretation, drafting of the manuscript and revision of the manuscript content, and takes responsibility for the integrity of the data analysis, and final approval of the manuscript. LS was responsible for data analysis and interpretation, drafting and revising the manuscript content, and final approval of the manuscript. HN, DS, XG, MB, NN and MCB contributed to data collection and analysis, revision and final approval of the manuscript. DV was responsible for data interpretation, revising manuscript content and final approval of the manuscript. All authors read and approved the final manuscript.
